# Fatigue Life Prediction of a SAE Keyhole Specimen as a Subcase of Certification by Analysis

**DOI:** 10.3390/ma17184521

**Published:** 2024-09-14

**Authors:** Xijia Wu, Zhong Zhang, Dany Paraschivoiu

**Affiliations:** Aerospace Research Center, National Research Council Canada, 1200 Montreal Rd., Ottawa, ON K1A 0R6, Canada; zhong.zhang@nrc-cnrc.gc.ca (Z.Z.); dany.paraschivoiu@nrc-cnrc.gc.ca (D.P.)

**Keywords:** fatigue, keyhole specimen, structural integrity, life prediction, certification by analysis

## Abstract

To advance the technology of Certification by Analysis (CbA), as called for by the aerospace industry, the fatigue problems of SAE keyhole specimens are analyzed to demonstrate a subcase of CbA. First, phenomena identification and ranking table (PIRT) analysis is performed. Second, modeling of the key phenomena is conducted, and finally, verification and validation with the experimental results are achieved. In particular, the elastic/elastoplastic stress distributions in the keyhole specimens are obtained using the finite element method (FEM). Plasticity correction for stress/strain at the notch root is made using the modified Neuber’s rule along with the Ramberg–Osgood equation. The low cycle fatigue (LCF) crack nucleation life is analytically predicted using the modified Tanaka–Mura model, a.k.a. the TMW model, given the material’s elastic modulus, Poisson’s ratio, Burgers vector, and surface energy, without the need for coupon fatigue data regression. The Tomkins equation is used to simulate plastic crack growth within the notch plastic zone. The above analytical life predictions are validated against the SAE keyhole specimen tests, becoming the first successful case of fatigue CbA at a sub-element level.

## 1. Introduction

Fatigue life prediction is a critical task for maintaining the structural integrity of engineering platforms. It is needed not only in the initial design analysis but also in the prognosis and health management of air/ground vehicles during service. Especially in recent years, modeling and simulation (M&S)/digital twin technologies and certification by analysis (CbA) are called for to ensure environmentally friendly and economically variable development and the operational safety of large transport aircraft/vehicles [1]. Currently, structural fatigue life is assessed and managed using two distinct approaches: (1) safe life and (2) damage tolerance. Safe Life assumes that there are no cracks in the structure during the specified operational life and is also called crack initiation life. The Damage Tolerance approach is quantified as the structural ability to endure crack growth from a detectable initial size to fracture (i.e., reaching the critical crack size corresponding to the fracture toughness of the material). Usually, there is a gap between physical crack initiation (in the range of a few to a hundred microns, depending on the material’s microstructure) and detectable crack size (in the order of millimeters, depending on the non-destructive inspection technique) in structures. This leaves a great uncertainty in the total fatigue life of engineering structures.

In the 1970s, the Society of Automotive Engineers (SAE) Fatigue Design & Evaluation Committee conducted a test program to provide a set of basic data for determining the validity of various fatigue life estimation and analysis methods. The test program used a keyhole specimen configuration to allow observation of both crack initiation and propagation, where crack initiation was arbitrarily defined at 2.5 mm. Once a crack originated at the notch root, it resembled a compact tension (CT) specimen for fatigue crack growth rate testing. Two steels, Men-Ten and RQC-100, commonly used in the automotive vehicle industry were incorporated. Data on the basic material properties, including monotonic and cyclic stress–strain curves and basic fatigue properties in terms of Coffin–Manson–Basquin (CMB) equation parameters, were generated for both materials. Constant amplitude tests were performed on the “component-like” specimen as basic test cases. In addition, variable amplitude fatigue tests were also conducted using three in-service loading profiles at several load levels. The details of the test program can be seen on the Altair/eFatigue website [2]. 

**Figure 1:**
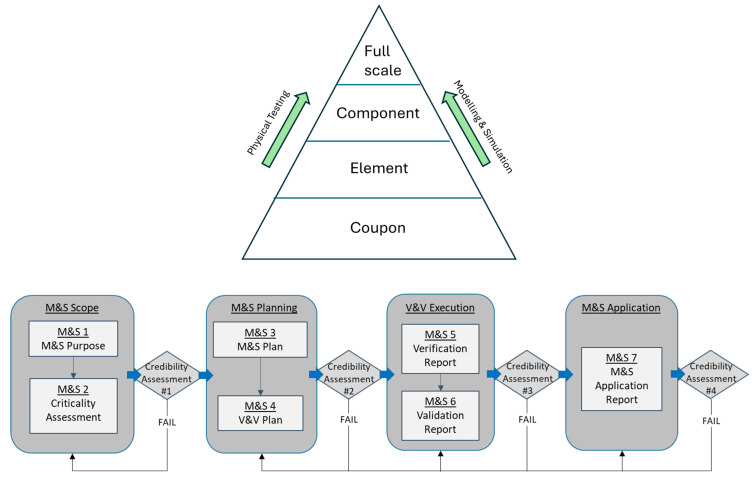
The airframe certification pyramid process and the credibility assurance framework for CbA.

For CbA of fatigue, one needs to select/develop the conceptual/mathematical/computational model with input values for the intended application, corresponding to the criticality level of the design. Then, verification/validation and uncertainty quantification need to be performed from the material coupon to the element and component, and even up to the full-scale structure levels in a pyramid block process [1], as shown in Figure 1. The SAE keyhole specimen represents material and structural features at the element level. It is suitable as a validation case for analytical fatigue life prediction. 

## 2. Literature Review

The conventional fatigue life prediction practice starts with fatigue coupon testing to collect a large amount of fatigue failure data (one datum per coupon) and then data regression is performed on the data using empirical equations, e.g., the Coffin–Manson–Basquin (CMB) equation [3,4,5] and the Morrow equation [6]. Prediction is usually made within the range of tested conditions. If the amount of data is statistically significant, the confidence level of the fatigue life can be determined, following the ASTM E739 Standard [7]. A number of empirical relationships have also been proposed to consider the effect of means stress or stress ratio R = σ_min_/σ_max_, such as Goodman [8], Soderberg [9], Smith, Watson, and Topper [10], Walker [11], etc., which are useful for analyzing fatigue under variable amplitude loading conditions. These relationships extend the basic zero-mean fatigue relationship, similar to the CMB equation, for general loading profiles that are often seen in real life. 

Many fatigue life prediction studies were conducted for the SAE keyhole specimen, using the results from the program as the test case for validation [12,13,14,15,16,17,18,19,20,21,22]. In the aforementioned studies, fatigue crack initiation life was predicted using the usual method of plasticity correction at the notch, i.e., the Coffin–Manson–Basquin (CMB) approach in combination with Neuber’s rule or the modified Neuber’s rule. This approach requires pre-determination of the CMB equation parameters, which are obtained from the regression of a large amount of experimental data generated at the material coupon level.

Recently, a pure analytical fatigue crack nucleation model has been developed by Wu [23], modifying the original Tanaka–Mura model [24]. This modified Tanaka–Mura model, thereafter called the TMW model, allows the determination of fatigue crack nucleation life by using the material’s basic physical properties, such as Young’s modulus, Poisson’s ratio, the Burgers vector, and surface energy, without the need for coupon data regression; therefore, it has the potential to reduce testing costs significantly. It has been applied to many metals and alloys including Type 316 stainless steel, low-alloy steels, aluminum alloys, titanium alloys, and Ni/Co-based superalloys [23,25]. Therefore, researchers are attempting to extend this basic material property relationship to higher levels in the CbA process.

This paper presents a computational fatigue life prediction approach for the SAE keyhole specimen with a fatigue crack of up to 2.5 mm. In contrast to the conventional fatigue crack initiation life prediction approach, the present method does not require breaking the fatigue specimen. All fatigue coupon and keyhole specimen test data serve as validation data. First, three-dimensional elastic-plastic finite element analysis is performed to validate the modified Neuber’s rule for plasticity correction. Second, the TMW is used to compute fatigue crack nucleation life with a crack size at the microstructural scale (0.1 mm, as adopted by most original equipment manufacturers (OEMs) [26]). Then, the Tomkins model [27] is used to compute plastic crack growth up to 2.5 mm under constant amplitude loading. Thus, this case study completes a pseudo-CbA of a structural element feature (notch).

## 3. Modeling Methods

**Figure 2:**
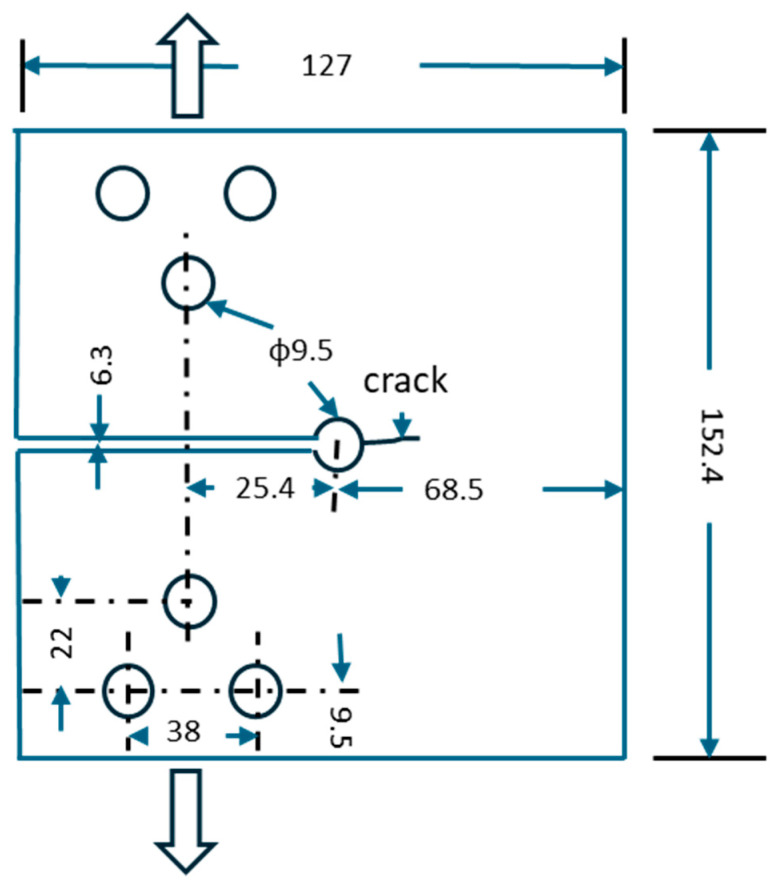
The keyhole specimen configuration with a thickness of 9.5 mm. All units are in millimeters.

The keyhole specimen was designed to have a circular hole at the end of a slot to permit studies of both crack initiation and crack propagation [2], as schematically shown in Figure 2. Loads were applied to the specimen/component using a mono-ball fixture with tightening bolts. The fixture allowed both tension and compression load to be transferred to the specimen. Therefore, the material at the notch root would experience typical low-cycle fatigue (LCF). Once a crack is formed through the thickness, the experimental setup resembles that of a CT specimen for fatigue crack growth rate testing.

Using phenomena identification and ranking table (PIRT) analysis, three important phenomena are considered in modeling and simulation:

1. Elastic-plastic stress–strain states at the notch in the specimen/element; a finite element method (FEM) model is used to validate the modified Neuber’s rule for plasticity correction.
2. Crack nucleation under LCF conditions.
3. Plasticity-driven short crack growth up to 2.5 mm.

### 3.1. Finite Element Analysis of the Keyhole Specimen and Plasticity Correction

**Figure 3:**
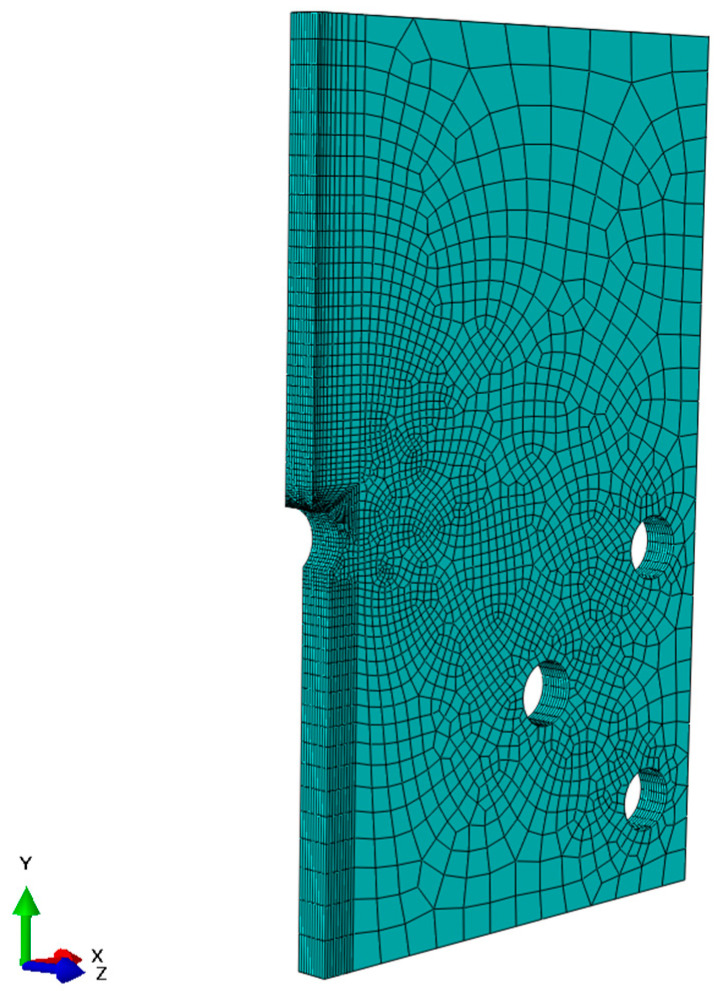
The FEM model of the keyhole specimen.

**Figure 4:**
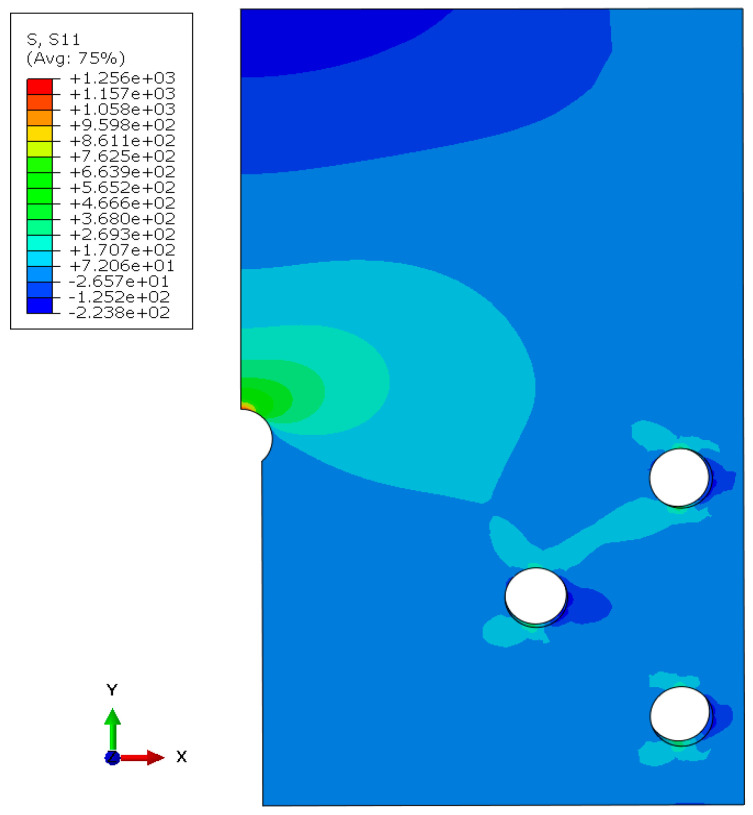
The stress contour maps for the keyhole specimen under 35.6 kN. The unit of stress is MPa.

**Figure 5:**
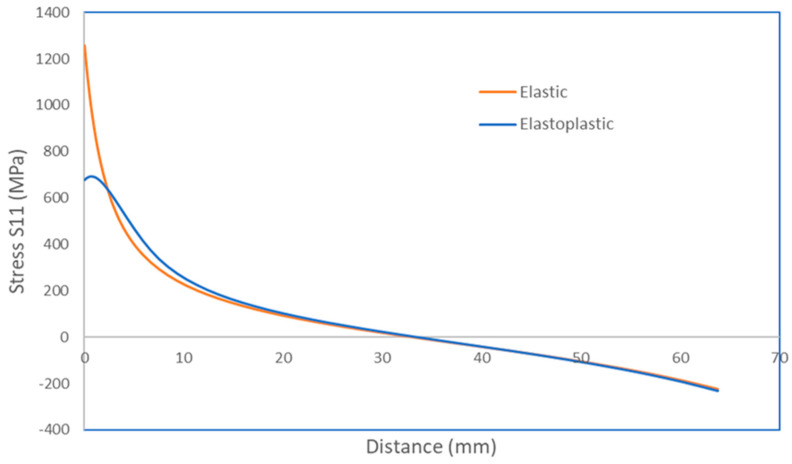
The elastic/elastoplastic stress as a function of distance from the notch under 35.6 kN.

A FEM model of the keyhole specimen is built in ABAQUS, as shown in Figure 3, where half of the specimen is shown by symmetry. The geometry model comprises 27,750 quadratic hexahedral elements (C3D20R) in Abaqus. Mesh refinement is applied near the notch with an average element size of 0.5 mm to accurately capture stress gradients. The meshes around the bolt holes are not refined because they are part of the fixturing and are not expected to be fatigue-failed. According to Saint Venant’s principle, the stress distributions around those bolt holes do not affect the stress distribution at the notch of interest. Both elastic and elastoplastic FEM stress analyses are performed. The elastoplastic simulations were conducted using Abaqus 2022, utilizing the built-in Ramberg–Osgood material model. The material properties of Men-Ten and RQC-100 needed for elastic-plastic analyses are given in Table 1, according to Refs. [2,13]. The stress contour in the keyhole specimen under a load of 35.6 kN is shown in Figure 4, and the elastic and elastoplastic stresses are compared along the distance ahead of the notch, as shown in Figure 5. Apparently, the keyhole notch has a strong stress concentration effect that causes material yielding in the region ~2.5 mm ahead of the notch. Normally, an elastoplastic stress analysis should be performed for each loading case. However, this would be very computationally costly, especially for a large component. Therefore, it is sensible to establish a plasticity correction rule that would convert elastic stress into true elastoplastic stress. This method would be very useful for variable amplitude loading analysis for fatigue evaluation under real service loading profiles.

In the elastoplastic analysis, the Ramberg–Osgood equation is employed to represent the cyclic stress–strain response:

(1) $$\frac{\Delta \varepsilon }{2}=\frac{\Delta \sigma }{2E}+{\left(\frac{\Delta \sigma }{2K^{\prime }}\right)}^{\frac{1}{n^{\prime }}}$$

where *K′* is the cyclic plastic strength and *n′* is the strain sensitivity exponent.

Newport and Glinka modified Neuber’s rule by equating the elastic energy at the maximum elastic stress to the elastoplastic energy under the Ramberg–Osgood curve [17]:

(2) $$\frac{{\left(\sigma_{max}\right)}^{2}}{2E}=\frac{\sigma_{a}^{2}}{2E}+\frac{\sigma_{a}}{n^{\prime }+1}{\left(\frac{\sigma_{a}}{K^{\prime }}\right)}^{\frac{1}{n^{\prime }}}$$

where $\sigma_{max}$ is the maximum stress by pure elasticity and $\sigma_{a}$ is the elastoplastic stress amplitude. 

Under the load of P = 35.6 kN, the elastic notch stress is *σ_max_* = 1255.7 MPa. The plasticity-corrected stress (amplitude) is determined by Equation (2) to be *σ_a_* = 658 MPa in RQC-100. The FEM-computed elastoplastic stress is 678 MPa. By comparison, the error is only 3%, which should be considered satisfactory for the benefit of simplified analysis. The elastic stresses of other loading cases are easily obtained by a factor of proportionality, *σ_max_* = 1255.7 × P/35.6 (MPa), and the plasticity correction can be obtained via Equation (2). 

### 3.2. Fatigue Crack Nucleation

**Figure 6:**
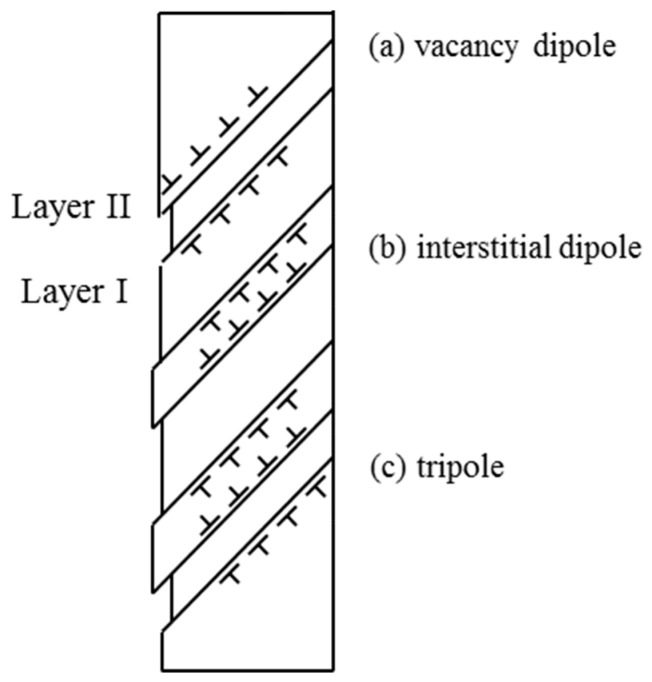
Dislocations in (**a**) vacancy dipoles (forming an intrusion), (**b**) interstitial dipoles (forming an extrusion), and (**c**) tripoles (forming an intrusion–extrusion pair) at the surface.

The conceptual model of fatigue crack nucleation is developed based on persistent slip band (PSB) formation under cyclic loading [28,29]. Dislocations emanating from PSBs can form intrusions and extrusions on the surface. Extrusions can be regarded as interstitial dipoles at the surface, while intrusions comprise vacancy dipoles, as schematically shown in Figure 6. Many scanning electron microscopy (SEM) observations have been made on crack nucleation at PSBs [30,31]. A fatigue crack nucleation model was originally developed by Tanaka and Mura, based on continuously distributed inverted dislocation pileup [24]; however, their original derivation led to a total plastic strain having a physical dimension of [m^2^]. Recently, Wu revised the Tanaka–Mura model using the true strain definition and obtained the following plastic strain-based formula (the mathematical model) for fatigue crack nucleation [23]:

(3) $$N_{c}=\frac{8\left(1-\nu \right)R_{s}w_{s}}{3\mu b}\frac{1}{{\Delta \varepsilon_{p}}^{2}}$$

where *N_c_* is the cycle number to crack nucleation, *w_s_* is the surface energy [32], *µ* is the shear modulus, *v* is Poisson’s ratio, *b* is the Burgers vector, and *R_s_* is the surface roughness factor (for an idealized smooth surface such as an electropolished surface, *R_s_* = 1; for machining surfaces, *R_s_* = 1/3 [23]).

**Figure 7:**
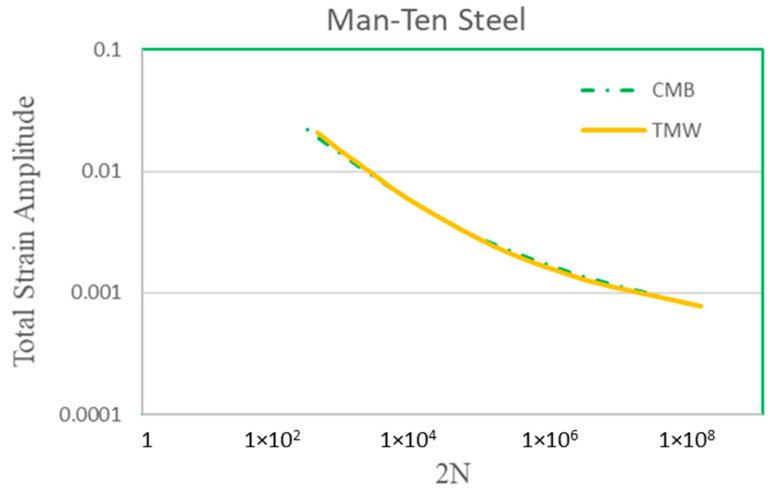
Theoretical prediction of Equation (3) in comparison with the Coffin–Manson–Basquin curve for Men-Ten steel.

**Figure 8:**
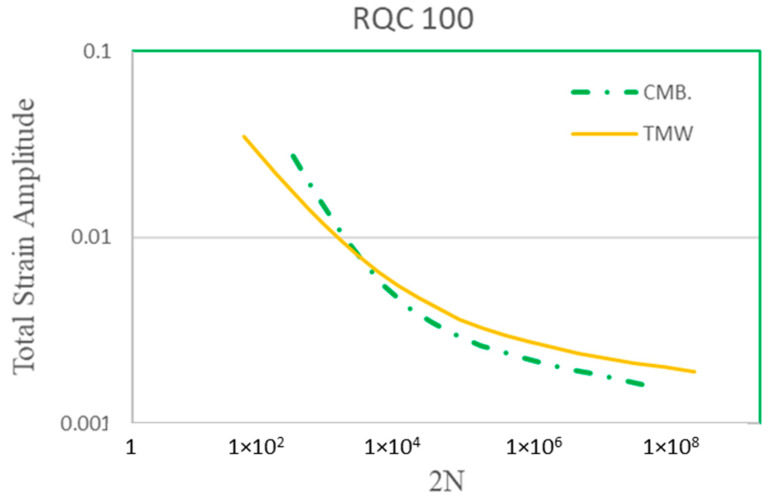
Theoretical prediction of Equation (3) in comparison with the Coffin–Manson–Basquin curve for RQC-100 steel.

The TMW model has been applied to many materials, including Type 316 stainless steel and low-alloy steels, aluminum alloys, titanium alloys, and Ni-based/Co-based superalloys [23,25]. For the interest of this study, it is further compared with the Coffin–Manson–Basquin relationships for Men-Ten and RQC-100 steels, as shown in Figure 7 and Figure 8, respectively. The material properties, as inputs for Equation (3), of the two steels are given in Table 2, where the Burgers vector and surface energy are for pure iron. The CMB curves are computed using the parameter values given in [2].

### 3.3. Short Crack Growth

In the keyhole specimen, when a crack was first formed at the notch root, it was well within the plastic zone under the test condition. Therefore, the keyhole notch root crack can be categorized as a plastically short crack. For the growth of fatigue cracks in a plastic region, Tomkins proposed a crack growth rate equation, in the same form as in [27]:

(4) $$\frac{da}{dN}=\frac{\pi^{2}}{8}{\left(\frac{\Delta \sigma }{2\sigma_{u}}\right)}^{2}\frac{\Delta \varepsilon_{p}}{\left(2n^{\prime }+1\right)}a$$

where *a* is the crack length, Δε_p_ is the plastic strain range, $\sigma_{u}$ is the ultimate tensile strength of the material, and $n^{\prime }$ is the strain sensitivity exponent. 

Equation (4) takes into account the effect of plastic strain at the notch root on fatigue crack growth, as plastic deformation is involved in LCF. All the parameter values in Equation (4) for the two steels are given in Table 1 as well. Integration of Equation (4) leads to

(5) $$N_{g}={\left[\frac{\pi^{2}}{8}{\left(\frac{\Delta \sigma }{2\sigma_{u}}\right)}^{2}\frac{\Delta \varepsilon_{p}}{\left(2n^{\prime }+1\right)}\right]}^{-1}\ln\frac{a_{i}}{a_{n}}$$

where *N_g_* is the cycle number of crack growth, *a_n_* is the crack nucleation size, in this case 0.1 mm according to the microstructural definition, and *a_i_* is the crack initiation size according to the engineering definition, which in this case is 2.5 mm. The stress and plastic strain ranges are evaluated by the Ramberg–Osgood equation.

## 4. Results

**Figure 9:**
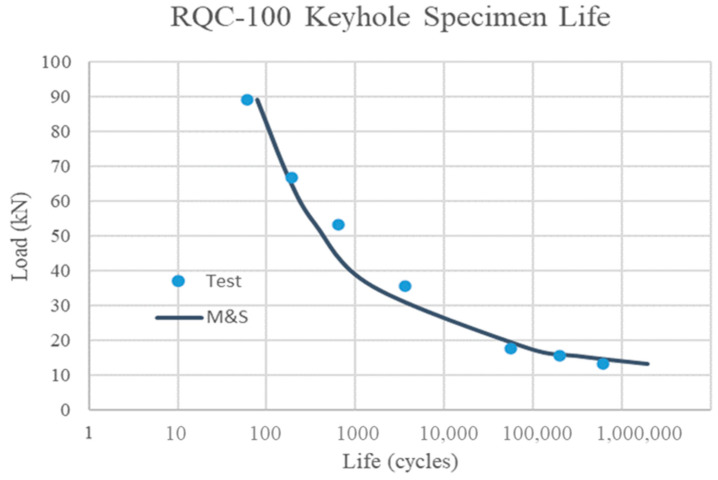
Comparison of keyhole specimen modeling and simulation (M&S) results with tests for RQC-100 steel.

The total life of the keyhole specimen with a crack grown to 2.5 mm is calculated as the sum of crack nucleation life given by Equation (3) and crack propagation life given by Equation (5), as shown in Table 3. The keyhole test results are taken from ref. [2]. The total life is compared with the experimental observation, as shown in Figure 9. The corresponding specimen ID of RQC-100, the elastic stress, the elastoplastic stress (by the modified Neuber plasticity correction), and the plastic strain amplitude are also given for reference. This study focuses on the cases of R = −1, since it represents the baseline fatigue property for the material. 

When the crack extends beyond 2.5 mm, its growth is controlled by linear fracture mechanics (LEFM). The LEFM fatigue crack growth rate is often expressed as the Paris equation (eFatigue) [2,33]:

(6) $$\frac{da}{dN}=5.2\times 10^{-9}\Delta K^{3.25}\;\;\left(\mathrm{mm}\cdot {\mathrm{cycle}}^{-1}\right)\;$$

where Δ*K* is the cyclic stress intensity factor range. 

**Figure 10:**
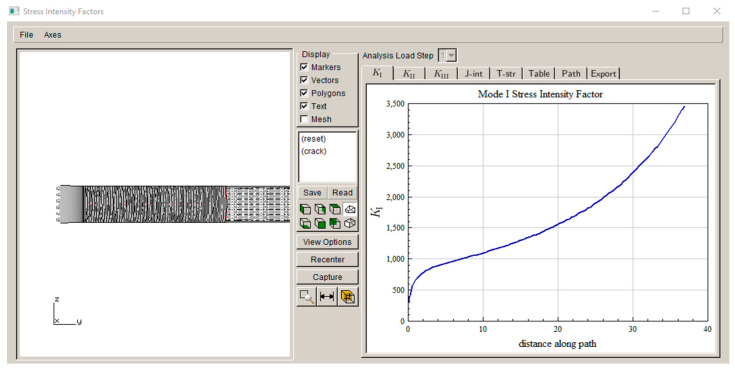
FRANC3D-simulated crack growth steps.

As an example, LEFM fatigue crack growth simulation under the load of 13.3 kN is conducted using FRANC3D. The simulated crack growth steps are shown in Figure 10 (Note: K_I_ is plotted with units of MPa√mm, as computed in accordance with the FEM geometry model unit), and the calculated crack growth life from *a_i_* = 0.1 mm to a fracture toughness of 109 MPa is 78,519 cycles (Note: only the positive load cycle is considered to drive crack growth). As the LEFM crack growth life is a little more than 1/10 of the crack initiation life even at the lowest load in the present case, other high-load cases are not simulated.

The essence of CbA is to increase confidence in operational safety by means of modeling and simulation with minimal physical testing. This study is the first attempt at computationally predicting the fatigue life of a sub-structural element without breaking a material fatigue coupon. As only the nominal material properties are available as input without statistical characterization, the analytical life prediction is presented here only for demonstration. The following discussion focuses on the verification and validation (V&V) of the analytical model in comparison with the SAE keyhole experiments with regard to the confidence of satisfying potential “certification” requirements.

## 5. Discussion

**Figure 11:**
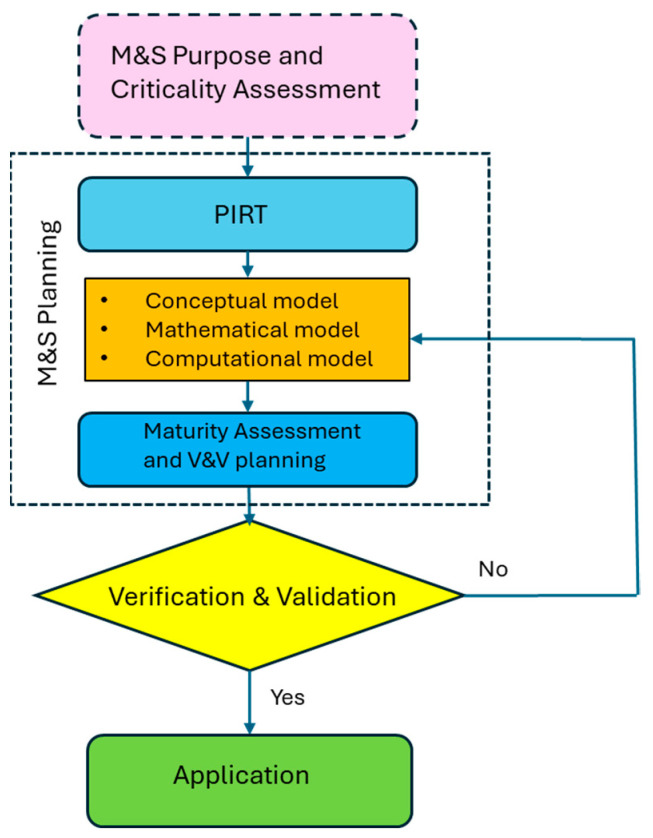
A generic process of modeling and simulation.

For the keyhole specimen case study, the PIRT analysis identifies three key phenomena: (i) the elastoplastic stress state at the notch root; (ii) crack nucleation under low-cycle fatigue conditions, and (iii) growth of the plastic short crack. The modified Neuber plasticity correction rule is combined with linear elastic FEM analysis to obtain the elastoplastic stress at the notch root. The simplest dislocation pileup-based model, i.e., the TMW model, is used to describe fatigue crack nucleation, and another simple crack growth model, i.e., the Tomkins model, is used for short crack growth within the notch root plastic zone. These conceptual/mathematical models are incorporated into ABAQUS as the computational model to simulate fatigue crack nucleation and propagation up to 2.5 mm. The modelling and simulation and verification and validation processes are schematically shown in Figure 11.

The prediction of the TMW model is compared with the Coffin–Manson–Basquin relationships (coupon behaviors) for Men-Ten and RQC-100, as shown in Figure 7 and Figure 8, respectively. Excellent agreements are found between the TMW model and the CMB equation, especially in the LCF regime (<10^4^ cycles). It should be emphasized that Equation (3) does not need the breaking of a fatigue coupon for calibration, so it is a class A prediction (forecast before the event occurs). Towards high-cycle fatigue (HCF) (>10^5^) at low strain amplitudes, a disparity between TMW and CMB is observed. The experimental scatter of fatigue has been known to be notoriously high for HCF. It is thought that the microstructure plays an important role in fatigue scattering. This issue is currently being addressed using microstructure-based fatigue modeling via representative volume elements (RVEs) for Haynes 282 [34]. Without the experimental raw data (not the CMB regression relationship), fatigue scattering cannot be analyzed further for these two steels in the present study.

In addition to crack nucleation, crack propagation life (to 2.5 mm) is added. The total fatigue life predictions for RQC-100 keyhole specimens are in good agreement with the test results, as shown in Table 3 and Figure 9. At low load levels, crack propagation only takes a small fraction of the total process life, which is perhaps no larger than the scatter itself. At high loads, crack propagation is a significant part of the total life, which is expected. The agreement with the experimental total life validates the TMW and Tomkins models as well. It is emphasized again that these models do not need the breaking of a fatigue coupon for calibration, so the total life prediction in this exercise is totally independent of a keyhole specimen test. However, it can be seen that the predicted life is longer than the experimental observations at low loads. This seems to be because the TMW prediction is larger than the best-fit CMB at low strain amplitudes. Further microstructure-based analysis may help to understand the scattering problem.

In this simulation analysis, plastic crack growth is assumed to proceed under constant stress and plastic strain amplitudes, which is approximately true given the plastic yielding in the notch root region, as shown in Figure 5. Beyond the plastic region, crack growth may be controlled by the parameters of linear elastic fracture mechanics (LEFM), e.g., the stress intensity factor range, ΔK. LEFM crack growth simulation from 0.1 mm to fracture is conducted using FRANC3D for the load case of 13.3 kN, and its propagation life is a little more than the plastic crack growth (to 2.5 mm) life measured using the Tomkins model and comparable to the experimental crack growth life of 85,500 cycles. At high loads, little of the total life is spent as LEFM cracks. Therefore, LEFM crack life is believed to be no larger than the scatter band of crack initiation in keyhole specimens. 

This keyhole specimen fatigue study opens an important question for potential certification by analysis, i.e., even though the basic crack nucleation and propagation rules established by coupon/analytical studies can be carried on up to the element level with structural features, uncertainty propagation is still in question as it involves the effects of the local microstructure, stress triaxiality, surface roughness, and possible residual stress induced by manufacturing. Traditionally, fatigue uncertainty quantification (UQ) is addressed through the testing of numerous samples to determine the probability of failure occurrence. An alternative approach is through microstructural fatigue simulation with real material grain size and orientation distributions as the material representative volume element [34]. The physics underlying Equation (3) can be easily incorporated into crystal plasticity analysis to identify crack-nucleating grain(s). Work of this nature for smooth coupons is currently underway in the authors’ laboratory. Of course, extensive simulation and physical testing are still needed to standardize the analytical procedures and data analysis to cover a wide range of microstructural scenarios and possible extremes for certification. This means “virtual testing” to augment the credibility and confidence levels from coupon to component, following the same rules of physics, to address structural life prediction and uncertainty quantification in CbA.

## 6. Conclusions

The fatigue of SAE keyhole specimens is studied as a subcase of CbA. Modeling and simulations are performed using analytical models, i.e., the TMW model for crack nucleation and the Tomkins model for plastic crack growth, without breaking fatigue coupons for calibration as the conventional method, e.g., the CMB equation, would need. From the CbA perspective, several salient points are emphasized as follows.

1. LCF crack nucleation life can be analytically predicted using the TMW model based on the applied plastic strain range, given the material’s elastic modulus, Poisson’s ratio, Burgers vector, surface energy, and surface roughness condition, without resorting to fatigue testing.
2. For structural applications, the cyclic plastic strain can be evaluated from the Ramberg–Osgood equation, and the modified Neuber’s rule can be used to perform plasticity correction around the structural features. This way, the TMW is augmented to the element level.
3. The Tomkins equation can be used to simulate plastic crack growth within the notch plastic zone.
4. The above analytical life prediction has been validated by SAE keyhole specimen tests under constant amplitudes with R = −1.

In summary, the present fatigue modeling and simulation approach has been shown to have a remarkable advantage over the conventional data regression approach in saving experimental efforts for CbA. For applications in real-life practical cases, it still needs to be explored for cases under variable amplitude loading at different stress ratios, which is beyond the current scope of this study. In addition, further uncertainty quantification needs to be established considering manufacturing microstructure variabilities.

## Figures and Tables

**Table 1:** Elastic-plastic properties of Men-Ten and RQC-100.

Materials	Man-Ten	RQC-100
Elastic Modulus, E (GPa)	203	203
Poisson’s ratio	0.3	0.3
Yield Strength, σ_ys_ (MPa)	325	565
Ultimate Strength, σ_u_ (MPa)	565	820
Cyclic Plasticity Strength K’ (MPa)	1200.6	1131.6
Cyclic Strain Sensitivity, n’	0.2	0.1

**Table 2:** Material Properties as input into Equation (3) for Steels.

E (GPa)	V	b (10^−10^ m)	w_s_ (J/m^2^)
203	0.3	2.48	2.37

**Table 3:** Keyhole specimen modeling and simulation (M&S) in comparison with tests.

Specimen ID	Load (kN)	Elastic Stress (MPa)	Elastoplastic Stress (MPa)	Plastic Strain (%)	Crack Nucl. Life, Equation (3)	Crack Growth Life, Equation (5)	Total	Exp. (a = 2.5 mm)	Exp. Cycles to Fracture
CR-1	13.3	469	451	0.010	1,864,312	51,230	1,915,542	605,000	690,500
CR-15	15.6	550.1	495	0.026	289,698	16,764	306,462	200,000	250,900
CR-14	17.8	627.7	528	0.049	79,684	7727	87,411	55,000	85,600
CR-13	35.6	1255.4	658	0.44	976	551	1527	3600	-
CR-17	53.4	1883	720	1.09	161	187	348	650	-
CR-12	66.7	2352	753	1.702	66	109	175	194	197
CR-18	89	3138	796	2.97	22	56	78	60	-

## Data Availability

The experimental data presented in this study are openly available in eFatigue: https://www.efatigue.com/, accessed on 1 May 2024.
